# Case Report: Immune dysregulation associated with long-lasting regression of a (pre)leukemic clone

**DOI:** 10.3389/fimmu.2023.1280885

**Published:** 2023-10-16

**Authors:** Joost B. Koedijk, Thomas B. van Beek, Marijn A. Vermeulen, Lennart A. Kester, Elizabeth K. Schweighart, Stefan Nierkens, Mirjam E. Belderbos, C. Michel Zwaan, Katja M. J. Heitink-Pollé, Olaf Heidenreich

**Affiliations:** ^1^ Department of Hemato-Oncology, Princess Máxima Center for Pediatric Oncology, Utrecht, Netherlands; ^2^ Department of Pediatric Oncology, Erasmus Medical Center (MC)/Sophia Children’s Hospital, Rotterdam, Netherlands; ^3^ Center for Translational Immunology, University Medical Center Utrecht, Utrecht, Netherlands; ^4^ Wolfson Childhood Cancer Research Centre, Newcastle University, Newcastle upon Tyne, United Kingdom

**Keywords:** spontaneous remission, acute myeloid leukemia, childhood, immune-mediated, case report

## Abstract

Regression of leukemia in the absence of disease-modifying therapy remains poorly understood, although immunological mechanisms are thought to play a role. Here, we present a unique case of a 17-year-old boy with immune dysregulation and long-lasting regression of a (pre)leukemic clone in the absence of disease-modifying therapy. Using molecular and immunological analyses, we identified bone marrow features associated with disease control and loss thereof. In addition, our case reveals that detection of certain fusion genes with hardly any blasts in the bone marrow may be indicative of an accompanying oncogenic fusion gene, with implications for disease surveillance- and management in future patients.

## 1 Introduction

Over the last two decades, studies in a variety of cancers have shown the potential of immune-mediated approaches to eradicate malignant cells (1, 2). However, not all cancer patients, including those with acute myeloid leukemia (AML), have benefited from this development (3, 4). The development of immunotherapy for this population is mainly hampered by the lack of tumor-specific antigens and the immunologically ‘cold’ tumor microenvironment (5, 6). Nonetheless, in rare cases, AML can regress in the absence of therapy and many reports have suggested that immunological mechanisms play a role (7). However, detailed immunological analyses at the moment of regression are lacking and therefore, the contribution of the immune system to such regressions remains unknown. Here, we describe the disease course and molecular- and immunological analyses performed at disease presentation, regression, and development of overt AML in a 17-year-old boy. The presented disease course suggests that the concept of immunoediting, including cancer elimination and immune escape, is applicable to the development of AML, and provides directions for future research (8). In addition, the retrospective identification of the *KMT2A*::*MLLT10* fusion gene at initial presentation, which was initially not detected by RNA-sequencing, is likely to change disease management in future patients.

## 2 Case description

**Figure 1:**
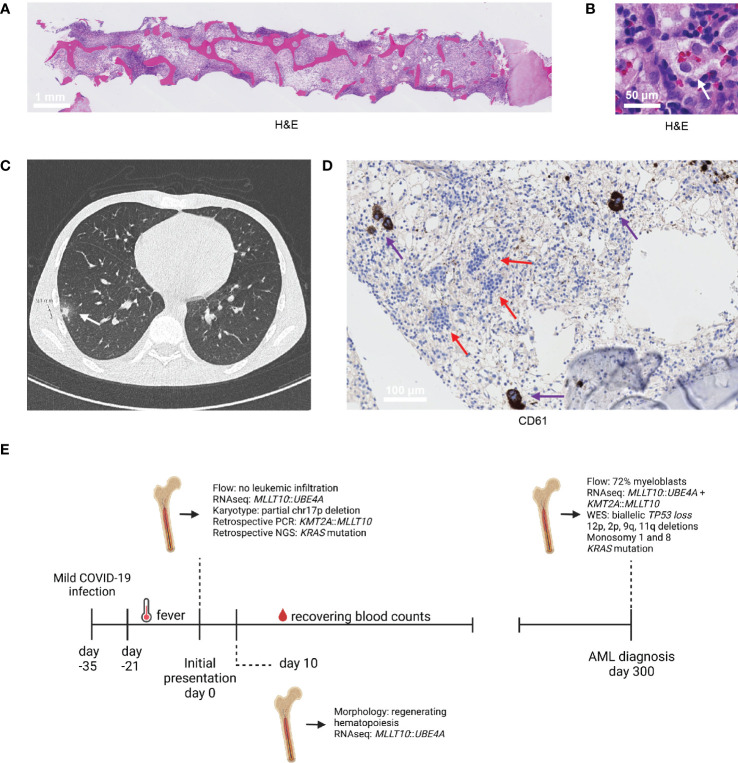
Diagnostic findings. **(A, B)** H&E section of the trephine biopsy at initial presentation **(A)** and an illustration of the hemophagocytosis present in this biopsy, indicated by the white arrow **(B)**. **(C)** CT scan of the thorax performed at initial presentation, which revealed two nodular lesions in the right lung. One nodular lesion with a diameter of 9 mm is shown (white arrow; the other lesion was similar and is not shown). **(D)** Immunohistochemistry stain of megakaryocytes (CD61, indicated by the purple arrow) in the trephine biopsy collected at 10 days from initial presentation. The clusters with blue cells, indicated by the red arrows, indicate recovering erythropoiesis. **(E)** Timeline of the most relevant diagnostic findings.

A 17-year-old boy without relevant medical history was referred to our center because of pancytopenia, hepatosplenomegaly, and non-remitting fever (≥3 weeks). Two weeks prior to fever onset, the patient had experienced a mild COVID-19 infection, which had resolved and for which he tested negative before the fever started. A bone marrow (BM) aspirate showed 21% activated monocytic cells (out of all BM cells; immunophenotype by flow cytometry: CD11b+, CD13+, CD14+, CD16+, CD34-, CD64+, HLA-DR+, IREM2+), neutropenia (<1%), and a prominent lymphocytic infiltrate (70% T cells, 5% B cells; normal immunophenotype), without evidence of leukemic infiltration. Furthermore, a BM (trephine) biopsy indicated hemophagocytosis, in line with the activated monocytic population that was detected (**Figures 1A, B**). These findings, in combination with increased ferritin- (986 μg/l; normal: 25-250 μg/l) and soluble IL-2 receptor (>55000 pg/ml; normal: 0-3000 pg/ml) blood levels, were compatible with immune dysregulation as seen in macrophage activation syndrome and hemophagocytic lymphohistiocytosis (HLH) (9, 10). Subsequently, a high-resolution CT-scan of the thorax showed two small nodular lesions with ground-glass opacity in the lower right lung, but a bronchoalveolar lavage and other diagnostic tests did not reveal any infectious pathogens (**Figure 1C**). Therefore, the COVID-19 infection before fever onset and/or a possible aspergillus were thought to be the most likely trigger(s) of the immune dysregulation. In the meantime, the boy was given erythrocyte- and thrombocyte transfusions and received empirical antibiotic and antifungal agents.

In the midst of the diagnostic process, a BM aspirate and trephine biopsy were repeated to follow-up on the initial findings (initial presentation +10 days). Fortunately, the BM aspirate and trephine biopsy showed regenerating hematopoiesis (**Figure 1D**). Furthermore, the monocytic population decreased in abundance, while the lymphoid infiltrate remained extensive. Because of these findings, no treatment for the immune dysregulation was started. Remarkably, routine bulk RNA-sequencing performed on BM aspirate material indicated the presence of a *MLLT10*::*UBE4A* fusion gene at initial presentation, albeit only 15 reads were detected. While the *KMT2A::MLLT10* fusion gene is common in both acute lymphoblastic leukemia (ALL) and AML, this *MLLT10*::*UBE4A* fusion gene had not been reported at that time (11). In addition, karyotyping revealed a partial deletion of chromosome 17p (*TP53*) in 1 out of 20 evaluated divisions. However, since there were no indications of oncogenic potential of the *MLLT10*::*UBE4A* fusion gene, only 1 out of 20 divisions showing a partial 17p-deletion, no leukemic blasts, and hematopoietic recovery, a wait-and-see approach was elected. Over the next weeks, the boy’s blood counts recovered to normal levels and the nodular lesions in his right lung decreased in size. He was followed up using differential blood counts every 2-3 months.

Nine months later, symptoms similar to those at initial presentation, apart from the fever, arose. A BM aspirate revealed 72% blasts (flow cytometry-based) with a monocyte-like morphology, but with a more immature immunophenotype compared to initial presentation (CD11b+, CD13+/-, CD14-, CD15+, CD16-, CD33+, CD34-, CD117-, CD123+, HLA-DR+, IREM2+/-). BM RNA-sequencing again revealed the *MLLT10*::*UBE4A* fusion gene, but this time in combination with the *KMT2A*::*MLLT10* fusion gene, and a diagnosis of AML was made. In addition to these fusion genes, biallelic loss of *TP53* due to a 17p-deletion and a *TP53* ^R248W^ mutation (VAF: 62%), deletions of 12p (*ETV6)*, 2p (*DNMT3A*), 11q (including *KMT2A* exon 10-36), and 9q, monosomy 1 and 8, and a *KRAS* ^G12C^ mutation (VAF: 89%) were identified by whole-exome sequencing. Retrospectively, a PCR and targeted sequencing on the trephine biopsy obtained at initial presentation indicated that the *KMT2A*::*MLLT10* fusion gene and *KRAS* ^G12C^ (VAF: 13%) mutation, respectively, had already been present at that time. Similarly, the detection of the 17p-deletion at AML diagnosis suggests that this alteration had already occurred at initial presentation. The boy received chemotherapy according to the NOPHO-DBH AML-2012 protocol, achieved complete remission, and was transplanted because of high-risk genetics one month ago. A timeline with the most relevant diagnostic findings is presented in **Figure 1E**.

## 3 Results and discussion

**Figure 2:**
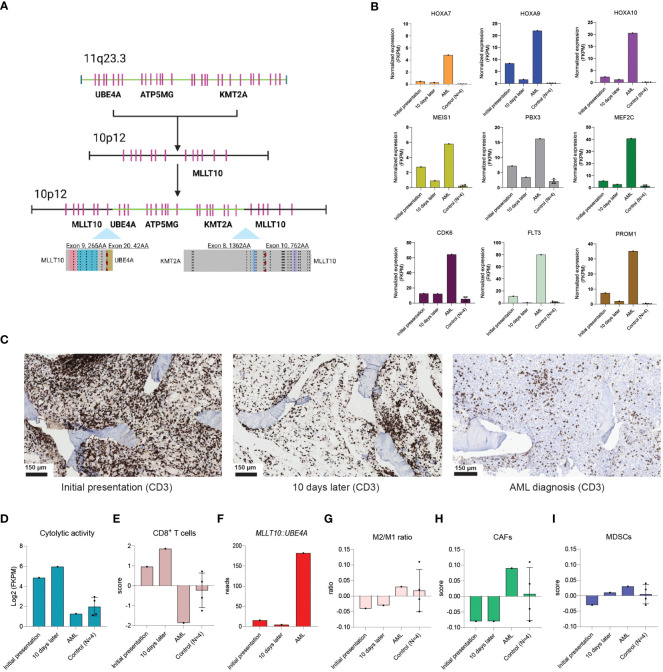
Immune dynamics at play at initial presentation, 10 days later, and at AML diagnosis. **(A)** Predicted mechanism of the single event leading to both the *MLLT10*::*UBE4A* and the *KMT2A*::*MLLT10* fusion genes. **(B)** Normalized gene expression (FKPM) of well-known downstream targets of *KMT2A*-rearrangements at initial presentation, 10 days later, and at AML diagnosis in bone marrow RNA-sequencing data from our patient, and compared to bone marrow RNA-sequencing data derived from 4 treatment-naïve children with early-stage rhabdomyosarcoma without malignant bone marrow infiltration (non-leukemic controls). **(C)** CD3 stains showing the T cell abundance in the trephine biopsy collected at initial presentation, 10 days later, and at AML diagnosis. **(D)** Illustration of the cytolytic activity score (geometric mean of *GZMA*, *GZMH*, *GZMM*, *PRF1*, *GNLY*) at the above-mentioned time points. **(E)** The estimated abundance of CD8^+^ T cells at the above-mentioned timepoints. **(F)** Illustration of the detected reads of the *MLLT10*::*UBE4A* gene at initial presentation, 10 days later, and AML diagnosis. **(G–I)** The ratio between pro(M1)- and anti(M2)-inflammatory macrophages **(G)**, cancer-associated fibroblasts (CAFs; H), and myeloid-derived suppressor cells (MDSCs; I) at initial presentation, 10 days later, AML diagnosis, and in the 4 non-leukemic controls.

This case is unique in several ways and provides valuable insights for clinical care and research. Retrospectively, the transcriptional orientation of both the *UBE4A* and the *KMT2A* gene suggests that the identified fusion genes were the result of a single event (**Figure 2A**). The detection of the one but not the other fusion gene at initial presentation may be explained by differences in promotor activity, illustrated by the more than 3-fold higher number of detected reads for *MLLT10*::*UBE4A* in comparison to *KMT2A*::*MLLT10* at AML diagnosis (182 versus 48 reads, respectively). In line with the presence of *KMT2A*::*MLLT10* at initial presentation, downstream targets of *KMT2A*-rearrangements such as *HOXA9*, *MEIS1*, and *PBX3* were upregulated at that time point in comparison to non-leukemic controls (4 pediatric patients with treatment-naïve early-stage rhabdomyosarcoma without malignant BM infiltration**;** **Figure 2B**). Accordingly, we postulate that a (pre)leukemic clone was present at that stage, which remained under control for 9 months before it developed into overt AML. Such (pre)leukemic clones may have a normal immunophenotype, complicating their detection using flow cytometry in case of low blast percentages. In future cases where a sole *MLLT10*::*UBE4A* fusion gene is detected with a low percentage or without any blasts, our case suggests that one should be aware that a concurrent *KMT2A*::*MLLT10* fusion gene and potentially a (pre)leukemic clone may be present as well. This is of particular relevance since children with *KMT2A*::*MLLT10* AML often show low blast percentages in the BM (12). If the *KMT2A*::*MLLT10* fusion gene is confirmed at disease presentation in cases similar to our patient (e.g., using DNA- and RNA-based PCR or FISH), frequent BM aspirates should be taken to monitor the blast percentage. If an elevated number of blasts is detected, treatment initiation should be considered as AML with defining genetic abnormalities may now be diagnosed with <20% blasts (12).

Furthermore, because of the extensive T cell infiltrate in the trephine biopsies collected at initial presentation and 10 days later, we performed immunogenomic analyses on BM RNA-sequencing data, which revealed a much higher cytolytic activity (geometric mean of *GZMA*, *GZMH*, *GZMM*, *PRF1*, and *GNLY*) and estimated abundance of CD8^+^ T cells (estimated using the Tumor Immune Dysfunction and Exclusion-framework) at both initial time points in comparison to when AML was diagnosed, and to non-leukemic controls (**Figures 2C–E**) (13, 14). Interestingly, the number of *MLLT10*::*UBE4A* reads and the expression of well-known *KMT2A*-related downstream targets decreased at 10 days after initial presentation, while the hematopoietic system showed signs of recovery (**Figure 1D**; **2B, F**). Therefore, we speculate that a specific immune response directed against the (pre)leukemic clone, in addition to the immune dysregulation affecting all blood lineages, was present in our patient. It is possible that the observed immune dysregulation and subsequent regression of the (pre)leukemic clone were related to the prior COVID-19 infection (15). Indeed, immune-inflammatory responses triggered by infectious pathogens may lead to anti-tumor immune responses via cross-reactivity of pathogen-specific T cells (16, 17). Furthermore, COVID-19 may also have acted as an oncolytic virus, resulting in the release of tumor antigens and priming of a tumor-specific immune response (18). Alternatively, leukemias themselves may also trigger such immune-inflammatory responses. For instance, several reports described HLH at disease presentation in various hematological malignancies (19, 20). Another study described a case of a 6-year-old girl with HLH that developed AML only 2 months after treatment for HLH was started, further supporting a role for immune-inflammatory processes in keeping (pre)leukemic clones in check (21). Nonetheless, disease control was lost over time. Indeed, despite a still substantial T cell infiltrate, cytolytic activity was markedly reduced at AML diagnosis (**Figures 2C, D**). Moreover, the estimated abundance of several immunosuppressive cell subsets (M2-/M1-like macrophage ratio, cancer-associated fibroblasts, and myeloid-derived suppressor cells) was increased at AML diagnosis, suggesting that the BM microenvironment had become more immunosuppressive over time (**Figures 2G–I**) (14). Consequently, we speculate that the additional genetic alterations identified at AML diagnosis led to immune escape of the (pre)leukemic clone (9, 22).

In conclusion, we present a unique case of long-lasting regression of a (pre)leukemic clone in the absence of therapy. Using molecular- and immunological studies, we identified BM features associated with regression suggesting immune-mediated disease control of AML. Accordingly, our case creates an impetus to identify tumor-reactive T cell receptors at the moment of regression, which we were not able to test due to the absence of viable material, since novel T cell receptor therapies for AML are urgently needed for AML. In addition, detection of the *MLLT10*::*UBE4A* fusion gene in a patient with a low blast percentage may indicate that a *KMT2A*::*MLLT10* fusion gene and a pre(leukemic) clone are present as well, with implications for disease management.

## 4 Patient perspective

This study was approved by the Institutional Review Board of the Princess Máxima Center for Pediatric Oncology (PMCLAB2021.207 & PMCLAB2021.238). Both the involved patient and the non-leukemic controls described in the text provided written consent for banking and research use of the specimens, according to the Declaration of Helsinki. Specifically, the described patient gave consent for publication of his medical history.

## Data availability statement

The original contributions presented in the study are included in the article/supplementary materials. Further inquiries can be directed to the corresponding authors.

## Ethics statement

The studies involving humans were approved by Institutional Review Board of the Princess Máxima Center for Pediatric Oncology (PMCLAB2021.207 & PMCLAB2021.238). The studies were conducted in accordance with the local legislation and institutional requirements. Written informed consent for participation in this study was provided by the participant/patient. Written informed consent was obtained from the participant/patient for the publication of this case report.

## Author contributions

JK: Conceptualization, Data curation, Formal Analysis, Methodology, Project administration, Writing – original draft. TvB: Formal Analysis, Writing – review & editing. MV: Conceptualization, Data curation, Formal Analysis, Methodology, Writing – review & editing. LK: Data curation, Formal Analysis, Methodology, Writing – review & editing. ES: Data curation, Writing – review & editing. SN: Methodology, Supervision, Writing – review & editing. MB: Conceptualization, Supervision, Writing – review & editing. CZ: Conceptualization, Methodology, Supervision, Writing – review & editing. KH-P: Conceptualization, Methodology, Supervision, Writing – review & editing. OH: Conceptualization, Funding acquisition, Methodology, Supervision, Writing – review & editing.
